# The Effect of Femur Positioning on Measurement of Tibial Plateau Angle: An In Vitro Study

**DOI:** 10.3390/ani12233419

**Published:** 2022-12-05

**Authors:** Alan Danielski, Miguel Angel Solano, Russell Yeadon

**Affiliations:** 1 The Ralph Veterinary Referral Centre, Marlow SL7 1YG, UK; 2 Department of Veterinary Medicine and Animal Sciences, University of Naples “Federico II”, 80138 Naples, Italy; 3 Lumbry Park, Alton GU34 3HL, UK

**Keywords:** TPLO, TPA, cranial cruciate ligament

## Abstract

**Simple Summary:**

Correct stifle positioning during radiographs has always been deemed vital to be able to reliably measure the tibial plateau angle (TPA) prior to surgical procedures. However, no veterinary study has so far quantified the effect of variation in limb positioning on the measurement of TPA with reference to the degree of femoral condyle superimposition. The aim of this in vitro study was to identify how different degrees of femoral hemicondylar superimposition affect the measurement of the TPA in normal stifles in dogs. A total of 176 radiographs were assessed by three different observers. Measurement of real TPA from radiographs with greater than 3 mm variation in femoral condylar superimposition is significantly affected by the malpositioning and it should therefore be interpreted with caution.

**Abstract:**

Five canine cadaveric pelvic limbs with intact cranial cruciate ligaments were used to quantify the effect of variation in limb positioning on the radiographic measurement of the tibial plateau angle (TPA) with reference to the degree of femoral condyle superimposition. Intra-osseous pin placement and a custom jig design allowed the controlled three-dimensional manipulation of limbs. Medio-lateral digital radiographic projections were taken with perfect femoral hemicondylar superimposition to establish a “reference” TPA (difference in position = 0 mm), and subsequently in varying degrees of supination/pronation and abduction/adduction. The lack of femoral hemicondylar superimposition for each radiograph was quantified using a tangential line technique with reference to the long tibial axis. A total of 176 radiographs were each assessed by three observers. “True” TPA was measured and it ranged within 17–25° across all limbs assessed. Variation in femoral condylar positioning ranged from −13 mm to +13 mm proximo-distally, and −11 mm to +11 mm cranio-caudally. Moreover, 3 mm non-superimposition of the femoral condyles produced 90.6% of measurements with 1° difference between measured and “true” TPA, and a sensitivity of 97.9% for a 2° difference. Further reduction in femoral condylar superimposition to 4 mm reduced the frequency of 1° difference between measured and “true” TPA to 84.9%, and to 94.8% for a 2° difference. In conclusion, measurement of TPA in large breed dogs from radiographs with greater than 3 mm variation in femoral condylar superimposition should be interpreted with caution.

## 1. Introduction

Cranial cruciate ligament (CrCL) rupture has been demonstrated to be one of the most common causes of lameness in dogs [1]. Numerous surgical techniques have been described for the treatment of cranial cruciate ligament rupture (CrCL) [2], and, in the past 25 years, the focus has shifted toward a paradigm of creating dynamic stability in the CrCL-deficient stifle by altering the bone geometry, typically employing tibial osteotomies [1]. Several authors have emphasized the importance of correct pre-operative measurement of the tibial plateau angle [3,4,5] for the planning of tibial osteotomy techniques or for the assessment of angular limb deformities via the application of the center of rotation of angulation (CORA) methodology [2].

Two methods have been described for the radiographic measurement of TPA. The conventional method, proposed by Slocum, uses the most cranial and the most caudal points of the medial tibial condyle as precise anatomical landmarks [6]. The tangential method instead uses a line tangential to the cranial, linear portion of the medial tibial condyle at the femorotibial contact point [7] or a line representing the common tangent of the two circles (one representing the joint surface of the femoral condyles in the articulating area and the other circle outlines the area of contact on the tibial plateau) and cut perpendicularly through the line between the midpoints of the two circles in the tibio-femoral contact point [8].

Determination of TPA is known to be influenced by a range of factors, including tibial and X-ray beam positioning [3,9], the presence of osteoarthrosis and associated osteophyte formation [4] and inter-observer variability [10]. Three different studies limited the maximum lack of superimposition between the femoral condyles on medio-lateral radiographs to 2 mm to minimize inaccuracies attributable to positioning [4,7,11]. Perfect superimposition of the femoral condyles does not assure true lateral positioning of the tibia in clinical patients, and it has been suggested that tibial positioning should therefore be evaluated with reference to superimposition of the tibial condyles, regardless of femoral positioning [3]. However, the same study remarked upon the difficulty of the precise identification of appropriate tibial anatomical landmarks, especially in the presence of osteophytosis, suggesting that superimposition of the femoral condyles might be a more reliable feature for the evaluation of positioning.

To the authors’ knowledge, no published study has specifically documented the influence of the lack of superimposition of the femoral condyles on the accurate measurement of radiographic TPA. Our hypothesis was that distal femoral positioning would affect TPA measurement and that a meaningful measurement of this malpositioning (which could significantly affect the measurement of the real TPA) could be established. The objective of this study is to identify how much the TPA changes relative to the position of the femoral condyles.

## 2. Materials and Methods

Five cadaveric hind limbs from large breed (25–35 kg) dogs (3 Greyhounds, 1 Lurcher and one Pointer) with intact cranial cruciate ligaments were used. The animals were euthanatized for reasons unrelated to this study. All limbs were considered free of orthopedic disorders and deformities, as confirmed by a pre-euthanasia clinical examination, post-euthanasia radiographic examination of all pelvic limb joints and gross examination of all major joint structures by dissection after completion of this study. The five legs were harvested by hemipelvectomy, including disarticulation of the sacro-iliac joint and pubic and ischial osteotomies, preserving all muscles associated with the femur, tibia and distal limb, including their insertions on the pelvis where appropriate. The limbs were then frozen at −18° and thawed at room temperature 24 h before radiography was performed. An 8 mm Steinman pin was inserted into the femoral medullary cavity in a closed fashion through the trochanteric fossa and advanced to reach the mid-third of the femoral diaphysis. Two 3.2 mm negative-profile, partially threaded (Ellis) pins were inserted into the greater trochanter and into the femoral neck, respectively, connected to the intramedullary pin by SK^TM^ ESF clamps (IMEX^TM^ Veterinary Inc., Longview, TX, USA). Two further 3.2 mm Ellis pins were inserted into the ilial body and ischiatic tuberosity, respectively, and were also connected to the intramedullary pin by SK^TM^ ESF clamps, creating a robust “handle” for the three-dimensional manipulation of the limb.

### 2.1. Limb Positioning and Radiographic Method

**Figure 1:**
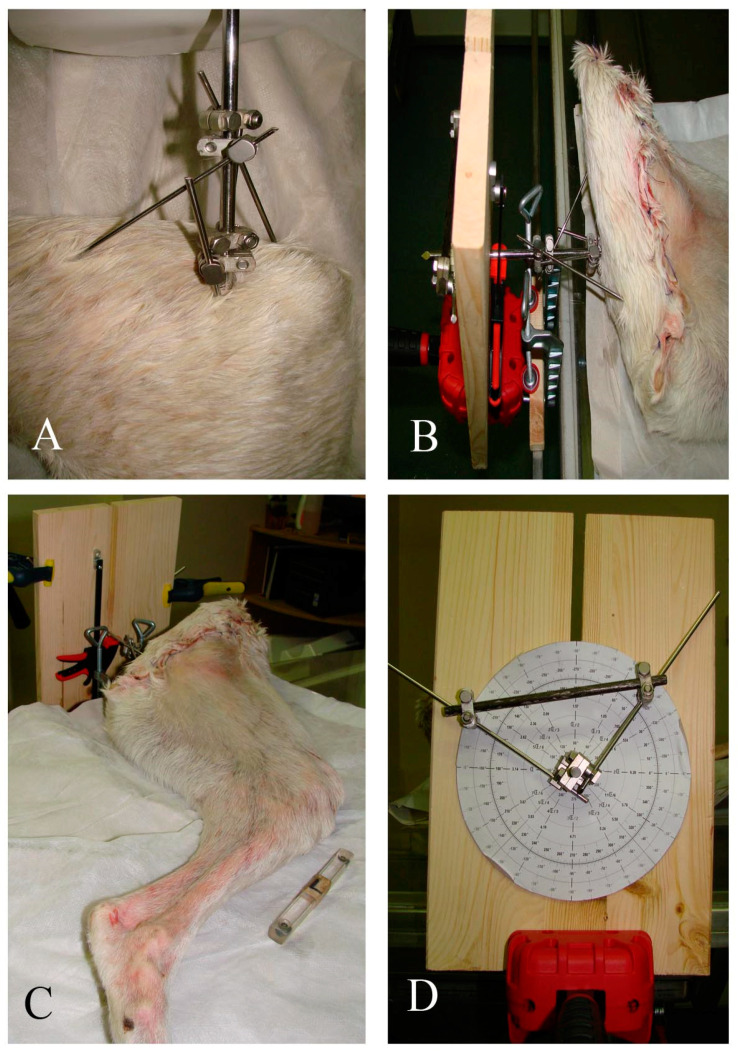
Limb positioning. Pins were used to secure the hemipelvis and to allow manipulation of the femur (**A**). The limb was subsequently connected to a table-mounted device (**B**,**C**) that allowed precise and controlled manipulation of the limb both in supination/pronation and in abduction/adduction (**D**).

Each limb was positioned in lateral recumbency and the intramedullary pin was connected to a custom-made jig designed to allow controlled manipulation of the limb both in supination/pronation and in abduction/adduction (Figure 1).

Medio-lateral radiographic projections were obtained with the primary beam centered over the tibial intercondylar tubercle in 135° of stifle joint flexion (assessed using a goniometer (14” Prestige Medical, Northridge, CA, USA) and manual palpation of anatomic landmarks). Collimation included the entire stifle joint, tibia and tarso-crural joint. A digital radiography system (Eklin Medical Systems Inc., Santa Clara, CA, USA) was employed. The initial radiographic position was established such that the two femoral condyles were perfectly superimposed, allowing the measurement of a “reference” TPA for each limb. Subsequent radiographs of the limbs were taken in a range of positions, with controlled variations in both supination/pronation and abduction/adduction, which was achieved by gradually turning the handle of the custom-made jig to the right and to the left, or by elevating or lowering the main pin that was inserted into the femur.

### 2.2. Radiographic TPA Measurement

**Figure 2:**
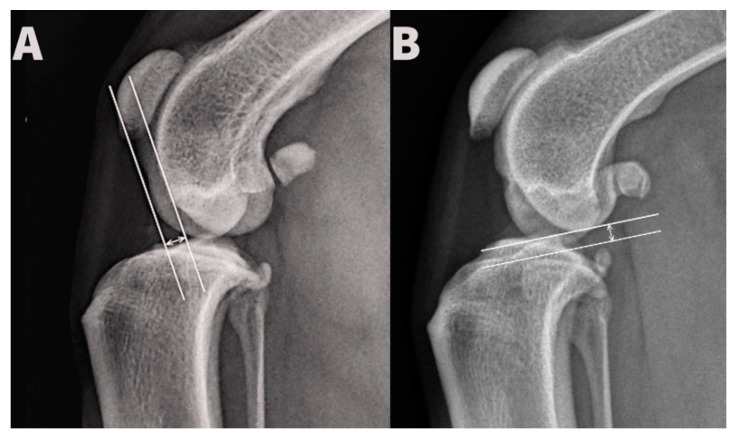
Measurement of the cranio-caudal (**A**) and proximo-distal (**B**) distance between the medial and lateral condyle.

Displacement of the femoral hemicondyles was established by three blinded observers for each radiograph. Cranio-caudal displacement was determined by drawing two lines parallel with the long tibial axis, aligned with most caudal aspects of the medial and lateral femoral hemicondyles, respectively, and measurement of the distance between them. Proximo-distal displacement was determined by drawing two lines perpendicular to the long tibial axis, aligned with the most distal aspects of the medial and lateral femoral hemicondyles, respectively, and measurement of the distance between them (Figure 2). Identification of the fossa of insertion of the tendon of the extensor digitorum longus and the more typically rounded shape of the lateral femoral hemicondyle allowed radiographic distinction between medial and lateral hemicondyles. All distances were measured in millimeters.

Proximal and caudal displacements of the lateral femoral condyle were recorded as positive values, while distal and cranial displacements were recorded as negative values. Measurements of both femoral hemicondylar displacement and TPA were performed directly on digital radiographic images using a commercial DICOM viewer (eFilm workstation 2.1, Merge^TM^ eMed, Eklin Medical Systems Inc., Santa Clara, CA, USA) and the software’s angle calipers. TPA was measured using the traditional method proposed by Slocum, drawing a first line along the tibial plateau connected the cranial and caudal margins of the medial tibial condyle and a second line along the tibial long axis, proximally through the mid-point of the tibial intercondylar eminences and distally through the mid-point of the talus. The angle of the line perpendicular to the tibial long axis and tibial plateau was defined as the measured TPA [12]. All observers used the same monitor and workstation. Three observers (surgeons with extensive experience in performing tibial plateau leveling osteotomy (TPLO) surgery) evaluated all radiographs using the conventional method proposed by Slocum [6]. For each limb, the “true” TPA was determined for each observer based on a consensus score from a minimum of three radiographs, with perfectly superimposed femoral condyles placed randomly within the sequence of radiographs for each limb. The “true” TPA was established separately for each observer for each limb. Inter-observer variability was not studied. The difference between the “measured” TPA of each radiograph and the “true” TPA was calculated and compared with femoral condyle positioning. The data were presented as histograms to facilitate their visual interpretation. A contour plot was used to represent an isoresponsive difference in TPA angle (in degrees) in a gradient of greens (light to dark green) with “proximo-distal femoral condyle non-superimposition (cm)” in the vertical axis (independent variable 1) and “cranio-caudal femoral non-superimposition (cm)” in the horizontal axis (independent variable 2). Minitab^®^ Release 14.20 statistical software was employed for data handling.

## 3. Results

A total of 176 radiographs were included in the study (limb 1:34 radiographs, limb 2:32 radiographs, limb 3:33 radiographs, limb 4:44 radiographs and limb 5:33 radiographs) to provide a total of 528 measurements between the three observers.

Femoral condylar positioning ranged from −13 mm to +13 mm in the proximo-distal direction (mean 0.0 mm, median 0 mm) and from −11 mm to +11 mm in the cranio-caudal direction (mean +0.9 mm, median 0 mm). The “true” TPA for all limbs ranged from 17° to 25°.

For radiographs with the femoral condyles perfectly superimposed, the consensus “true” TPA was recorded in 83.3% of limbs (Table 1), while no limb was measured as being more than 1° different from the “true” TPA.

As the degree of femoral condylar superimposition was reduced, there was a general trend toward increasing variation in the difference between “measured” and “true” TPA and a resultant decrease in sensitivity, although the effect of inappropriate positioning did not appear to be consistent, with similarly malpositioned radiographs having a “measured” TPA either higher or lower than the “true” TPA by the same or other observers. The difference between “measured” and “true” TPA ranged from 0° to 9° across all radiographs.

**Figure 3:**
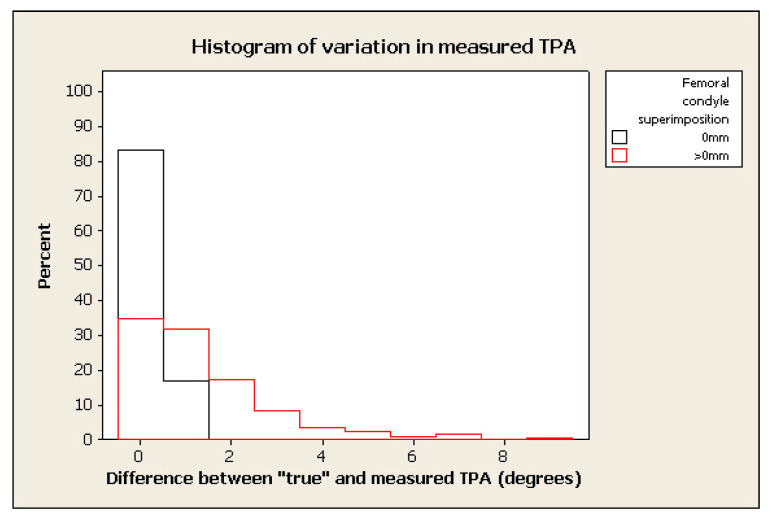
Histogram of frequency of measurements of TPA measured with femoral superimposition with 0 mm in difference between “true” and measured TPA in black and >0 mm in red.

**Figure 4:**
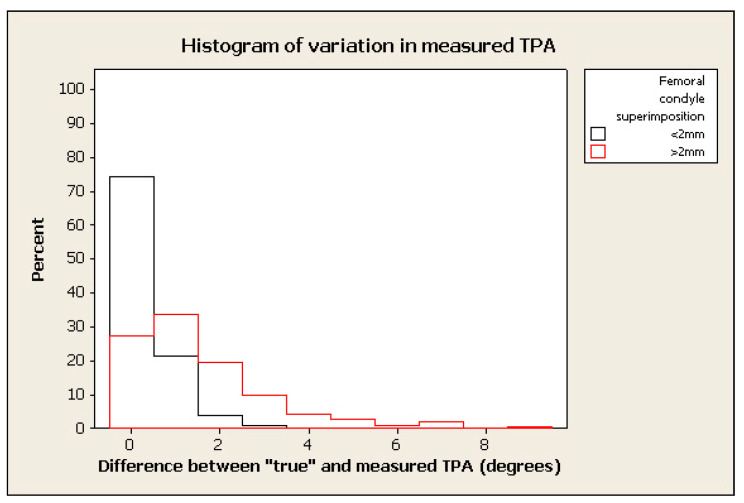
Histogram of % of frequency of measurements of TPA measured with femoral superimposition with <2 mm in difference between “true” and measured TPA in black and >2 mm in red.

**Figure 5:**
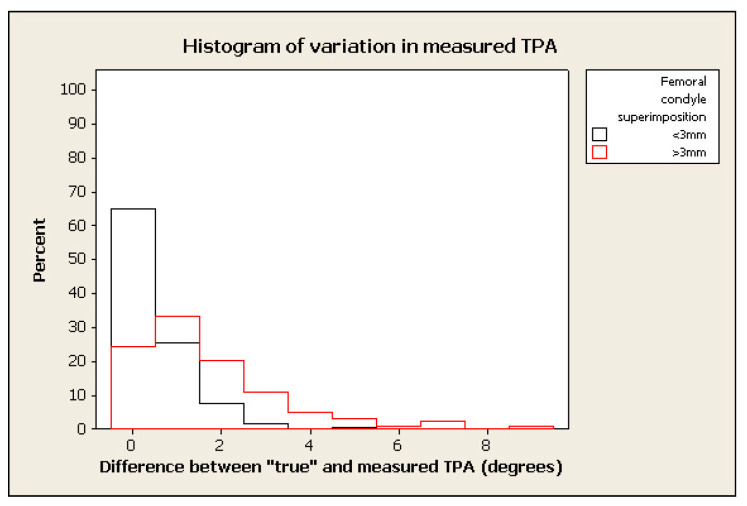
Histogram of % of frequency of measurements of TPA measured with femoral superimposition with <3 mm in difference between “true” and measured TPA in black and >3 mm in red.

**Figure 6:**
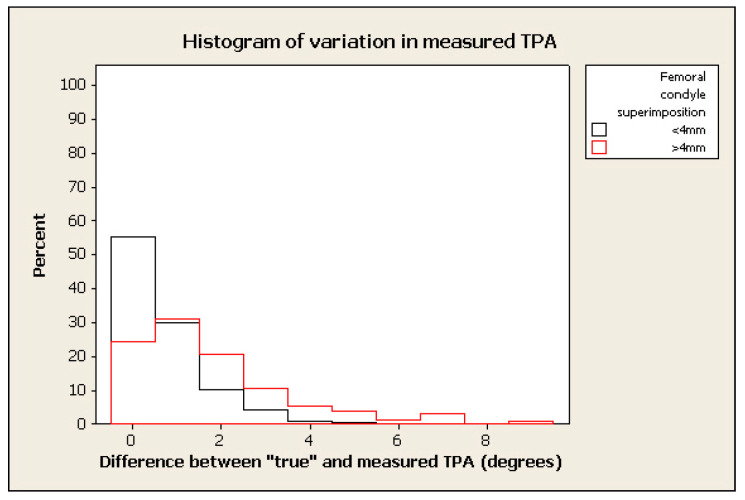
Histogram of % of frequency of measurements of TPA measured with femoral superimposition with <4 mm in difference between “true” and measured TPA in black and >4 mm in red.

The frequency of measurement remained relatively high where the tolerated difference between “true” and “measured” TPA was set to 2°–3°, in spite of marked malpositioning (see examples of histograms for % change in figures below) (e.g., frequency > 70% within 2° of “true” TPA for radiographs positioned with 0.4 mm of femoral condylar non-superimposition). (Figure 3, Figure 4, Figure 5 and Figure 6).

**Figure 7:**
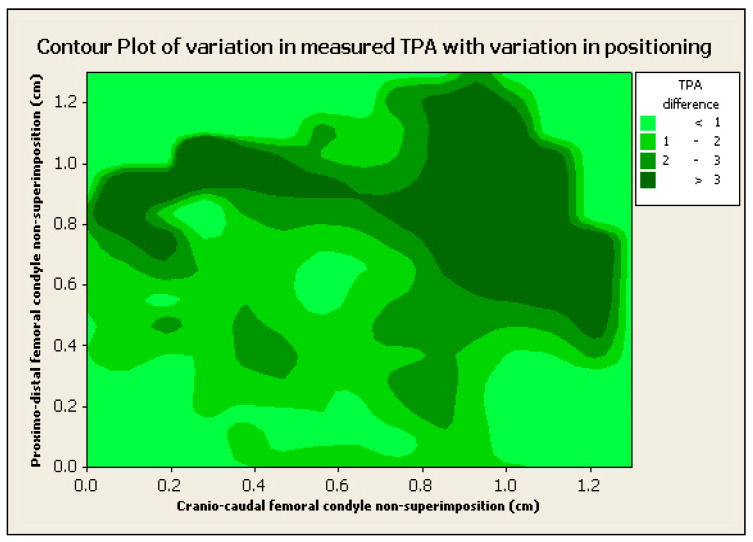
Contour plot of 3 dimensions: TPA angle difference (in degrees) with a gradient of greens, proximo-distal femoral condyle non-superimposition (cm) in the vertical axis and cranio-caudal femoral non-superimposition (cm) in the horizontal axis. Notice the general trend of a higher difference in TPA between “true” and measured TPA with increased non-superimposition in both planes (proximo-distal and cranio-caudal).

The effects of cranio-caudal and proximo-distal femoral condylar non-superimposition could not be reliably separated and no general trends were noted, with the exception of increasing variability with increasing malpositioning, as above (Figure 7).

## 4. Discussion

The importance of maintaining femoral condylar separation ≤ 2 mm, caudally, distally or cranially, has frequently been emphasized [4,7,11]. Five canine cadaveric hind limbs mounted on a custom jig mechanism were used in this study to quantify the effect of variation in limb positioning with reference to the degree of femoral condyle superimposition on the measurement of TPA in dogs with an intact cruciate ligament. In our study, 3 mm non-superimposition of the femoral condyles produced 90.6% frequency of measurements with a 1° difference between the measured and “true” TPA, and a sensitivity of 97.9% for a 2° difference. Further reduction in femoral condylar superimposition to 4 mm reduced the frequency of measurements with a 1° difference between measured and “true” TPA to 84.9%, and to 94.8% for a 2° difference. 

Determining the correct TPA before planning tibial osteotomy techniques is essential. Over- or under-rotation of the proximal tibial bone segment during the TPLO procedure, for example, can result, respectively, in caudal tibial thrust (increasing the biomechanical stress applied to the caudal cruciate ligament) and in the non-resolution of clinical dysfunction (because cranial tibial thrust is not neutralized). Determination of the precise TPA requires the observer to subjectively select precise landmarks from lateral radiographs of the tibial plateau and the hock. This process can introduce some variability, such as the positioning of the limb with respect to the X-ray beam, inter- and intra- observer variability, the type of film used (printed or digital), the method used to calculate the TPA, the presence or not of degenerative joint disease and osteophyte formation.

Numerous studies have sought to identify the most accurate method to determine TPA, standardizing possible subjective components [3,7,9,11]. Several measurement techniques have been proposed, each with relative proposed advantages. The use of the conventional method is controversial: in one study, the conventional TPA method seems to accurately represent the slope of the tibial plateau in normal stifle joints [11], and in another, it seems to underestimate the anatomic TPA (it is considered non-representative of the proximal articulating surface of the tibia) [7]. The same study remarks upon the accuracy of the tangential method, able to accurately determine the true orientation of the medial tibial condyle at the femorotibial contact point, therefore reproducing a more reliable shape of the main proximal articulating surface of the tibia [7]. However, using the central portion of the tibial plateau, as in the tangential method, has been proven to increase the inter-observer variability (especially when degenerative joint disease is present) [3].

In this study, radiographs were taken with the X-ray beam positioned over the stifle joint, in order to minimize the variability associated with the relative beam position. Previous studies have centered the beam on the tibial diaphysis [5,10], but this can lead to the perception of significant differences in TPA magnitude (mean TPA overestimation of 3.6° relative to beam centered on the stifle joint) due to unequal magnification and distortion of the tibial plateau [3]. A significant difference was not found between anatomic and radiographic TPA with the beam centered over the stifle and superimposition of the tibial condyles [3]. A novel split image radiographic technique has been also proposed to attempt the more accurate identification of the landmarks required for the measurement of TPA by collimating the beam on the stifle and hock joints separately [9].

Osteophyte formation, particularly on the caudal tibial plateau, can contribute to TPA measurement error and may increase inter-observer variability [3,4,9]. Limbs employed in this study were harvested from dogs unaffected by orthopedic disorders precluding the distortion of anatomical landmarks by degenerative changes and therefore reducing possible variabilities. Our study did not examine inter- and intra-observer variability, as it has already been adequately documented, with inter-observer variability reported as 0.8–2.4° and intra-observer variability as 1.5–1.7° [4,10]. While, in one study, it has been documented that the inter-observer variability is significantly improved when the observer has increased experience in measuring the TPA, in a second study, the level of experience of the observers did not seem to significantly affect the level of inter-observer variability [4,10].

It has been proposed that TPA measurements made using digital radiography, even if a significant difference was not found, can more closely approximate anatomic measurements than those made using printed radiographs, because of the ability to magnify the proximal portion of the tibia and adjust the radiographic exposure to optimize the identification of specific anatomic landmarks [5,7]. Direct digital radiography and the application of computer software measurement programs in this study may have helped to reduce the variability. Furthermore, calibration of the measuring tools intrinsic to the direct digital system employed assured accurate cross-referencing to published rotation charts (Slocum), which had originally been calibrated using a conventional radiographic film, which represented another potential source of error [5].

A major limitation of our study is that no limb was affected by pathology of the cranial cruciate ligament. CrCL rupture allows internal tibial rotation and may influence the tibial condyle positioning when applying varying degrees of supination/pronation and abduction/adduction to the leg. A previous study remarked upon the importance of tibial positioning, reporting that, in specimens from normal individuals, superimposition of the tibial condyles normally coincides with that of the femoral condyles [3]. Furthermore, CrCL rupture has also been demonstrated to allow a mean cranial tibial translation ± SD of 14 ±2.3 mm, which could artefactually increase the magnitude of TPA measurement [13]. It would be interesting to look at this variable in the future, to quantify the effect of variation in limb positioning correlated with femoral condyle superimposition on the measurement of the TPA of dogs with CrCL rupture. Furthermore, the range of true TPA reported in this study (17–25 degrees) is considered not representative of the true TPAs of dogs with rupture of the cranial cruciate ligament, which usually tends to be steeper. A second limitation of this in vitro model is represented by the fact that all muscles associated with the femur, tibia and distal limb, including their insertions on the pelvis, were appropriately preserved to simulate the clinical scenario as accurately as possible, and some variables, such as rigor mortis or variable muscle tension, may have partially affected our results.

## 5. Conclusions

In conclusion, our results suggest that measuring the TPA in stifles with femoral condyle superimposition < 3 mm is acceptable for clinical application when measuring the tibial plateau angle of dogs with intact cranial cruciate ligaments.

## Figures and Tables

**Table 1:** Frequency of results of various radiographic positions as determined by degree of femoral condylar superimposition, for range of differences between “measured” TPA and “true” TPA.

	Difference between “Measured” TPA and “True” TPA				
Femoral Condyle Superimposition	0°	1°	2°	3°	4°
**0 mm**	83.3%	100%	100%	100%	100%
**≤1 mm**	75.6%	94.4%	98.9%	100%	100%
**≤2 mm**	74.2%	95.5%	99.2%	100%	100%
**≤3 mm**	65.1%	90.6%	97.9%	99.5%	99.5%
**≤4 mm**	55.2%	84.9%	94.8%	98.8%	99.6%

## Data Availability

Not applicable.
